# Evaluating reflective practice groups in a mental health context: Swedish translation and psychometric evaluation of the clinical supervision evaluation questionnaire

**DOI:** 10.1186/s12912-019-0326-2

**Published:** 2019-01-31

**Authors:** S. Gabrielsson, Å. Engström, S. Gustafsson

**Affiliations:** Department of Health Sciences, Luleå University of Technology, SE-971 87 Luleå, Sweden

**Keywords:** Reflective practice, Clinical supervision, Mental health, Psychometric evaluation

## Abstract

**Background:**

Implementation of reflective practice groups in psychiatric and mental health contexts might improve the quality of care through promoting self-awareness, clinical insight, and facilitating stress management and team building. There is a need for valid and reliable instruments to test the outcomes of reflective practice groups in the mental health context. This study aimed to test the validity and reliability of the Swedish version of the Clinical Supervision Evaluation Questionnaire.

**Methods:**

The instrument was translated from English to Swedish using a translation and back-translation procedure. Data for the calculation of content validity was collected from an expert group. Data for the reliability analysis was collected from rehabilitation assistants and ward managers participating in reflective practice groups (*n* = 20). Content validity was measured by computing a content validity index. Construct validity was assessed by calculating the corrected item-total correlation statistics. Reliability was evaluated by analysing the Cronbach’s alpha coefficient, the intraclass correlation coefficient and inter-item correlations.

**Results:**

The content validity index for the scale as a whole was 0.94. Item-total correlations ranged between 0.23 and 0.81, and deletion of an item did not notably improve Cronbach’s alpha. Cronbach’s alpha for the scale was 0.89. The intraclass correlation coefficient for single measures was 0.35. The mean inter-item correlation was .37.

**Conclusion:**

The Swedish version of the Supervision Evaluation Questionnaire has a degree of reliability and validity that is comparable to the original version in English, indicating that it can be used as an assessment of reflective practice groups in the mental health context.

## Introduction

Person-centred care is increasingly considered the hallmark of mental health care [1]. Expectations are that mental health staff, regardless of the level of professional or vocational training, should be able to recognize and adapt to the individual needs of patients and service users [2]. This is cause for an enhanced interest in mental health care as a reflective practice [3] and thus a need for evaluating reflective practices in mental health.

## Background

While arguably a core competency of mental health professionals, the effectiveness of reflective practice remain unclear [4, 5]. Positive results from implementing RPGs have been reported suggesting that RPGs might promote self awareness, clinical insight and quality of care [6, 7], and also facilitate stress management and team building [6].

The Clinical Supervision Evaluation Scale (CSEQ) aims to assess staff perspectives on the process and impact of clinical group supervision [8]. It is intended to be “short and easy to complete” so that it can be used both in research and practice evaluation [8]. Arguably the widely used Manchester Clinical Supervision Scale (MCSS) [9, 10] is limited in this aspect as the number of items might limit its practical applicability in clinical settings; while the CSEQ has 14 items the original MCSS has 34 [9] reduced to 26 in the revised version [10]. In a psychometric evaluation of a Swedish translation the MCSS failed to exhibit satisfactory validity and reliability [11]. According to Horton et al. [8] the MCSS relates to “very particular supervision approaches” including a single supervisor model of supervision in which the supervisor offers advice rather than facilitates supervisees finding their own solutions through reflection. While clinical supervision lacks an agreed definition [12, 13] it often refer to group supervision led by a qualified supervisor and as something apart from managerial supervision. This is not necessarily the case with reflective supervision which is a reflective practice aimed at developing reflective capacity through professional supervision or as an element of workplace supervision [5]. Reflective supervision “is characterised by a collaborative partnership or group in which one person is typically more experienced than the other(s) but holds no authority, or power” [5]. Dawber [14] (2013a) suggests a peer facilitated model for RPGs, in which the primary function of facilitation is to balance opposing forces in the group by addressing resistance and promoting a sense of safety. The CSEQ was specifically designed to comply with a “non-managerial peer group” type of supervision [8] and as it is “designed to evaluate group supervision that utilises a facilitative approach to encourage reflection” the CSEQ has been proposed to be especially suited for evaluating RPGs [7]. To conclude: while other established evaluation tools for clinical supervision exists, most notably the MCSS, the specific features of CSEQ suggest it might be a valid and reliable alternative for evaluating RPGs.

Mental health nursing staff describe discussion and reflection on practice with colleagues as a vital source of support, validation, learning, hope, energy, and creativity [15]. Reflective practice is considered as facilitating the integration of theory and practice, a requisite for personal and professional development, and fostering person-centred approaches to care [16]. Because situations in practice do not always correspond neatly to the categories of theory, professional practice is not the straightforward application of theory to practice in a linear process [17]. Being professional is having the ability to adapt practice to the situation at hand, especially in situations of “uncertainty, uniqueness and conflict” ([18], p. XI). This is done by challenging the initial understanding of the situation, constructing a new understanding, and testing it – a process called *reflection-in-action* [17].

Professionals may also engage in *reflection-on-action*. By reflecting on their own practice, health care professionals can learn from experience and develop their ability and willingness for reflection-in-action [19]. Thus, reflective practice is believed to be supported by various reflective practices, e.g. reflective clinical supervision, self-reflection, group reflection and reflective writing. A Reflective Practice Group (RPG) is one form of reflective practice that has been developed and tested in the context of mental health nursing [6, 7, 14]. Dawber [14] describes RPGs as facilitated group supervision promoting reflection focusing on the interpersonal aspects of care delivery, allowing participants to share insights relevant to nursing practice in a supportive environment.

### Aim

The implementation of RPGs in psychiatric and mental health contexts might have beneficial outcomes for both staff and patients. To evaluate and further develop reflective practices, there is a need for sound and practical instruments targeting the process and impact of such practices. This study aimed to test the validity and reliability of the Swedish version of the Clinical Supervision Evaluation Questionnaire (S-CSEQ).

## Methods

### Context

Data for the reliability analysis was collected in conjunction with RPG sessions involving professional caregivers working in supported housing for persons with psychiatric disabilities in Northern Sweden. Rehabilitation assistants and unit managers at two housing units were offered to participate in a total of 12 RPG sessions over a period of 24 weeks. Each RPG session lasted for 90 min and involved a maximum of nine participants. The RPGs were facilitated by a registered nurse specialized in psychiatric care and conducted as part of an intervention aimed at promoting reflective practice and recovery-oriented care. Structured around the phases of the reflective process as described by Rodgers [20] and the process of care as described by Looi et al. [21], each session focused the needs of a specific service user and aimed to promote positive relationships, identify users’ resources and agree on recovery-oriented actions and approaches building on these. A detailed description and full evaluation of the intervention, involving both quantitative and qualitative data, will be reported elsewhere.

### The instrument

The CSEQ measures overall staff perception of clinical supervision in group supervision models which emphasize reflective process [8]. The CSEQ consists of 14 items related to three factors: the Purpose, Process, and Impact of clinical supervision (Table 1). Participants are asked to rate their agreement with 14 statements using a five-point Likert scale that ranges from ‘strongly agree’ (+ 2), ‘somewhat agree’ (1), ‘no opinion’ (0) ‘disagree’ (− 1) to ‘strongly disagree’ (− 2). Horton et al. [8] tested the CSEQ and found it satisfactory with regard to instrument validity and reliability.

**Table 1:** Factors and items in the Clinical Supervision Evaluation Questionnaire (CSEQ) (Horton et al., 2008)

Factors	Items
Purpose	1. The purpose of Clinical Supervision (CS) is to improve client care
	2. The purpose of CS is to enable clinicians to feel confident in their own practice
	3. I am clear about what I want to get out of CS
Process	4. I feel safe sharing clinical issues in supervision sessions
	5. There are well-established ground rules in my group
	6. I believe that any confidences I share are respected
	7. There is mutual trust between the members in my group
	8. I feel confident about bringing issues to CS
Impact	9. Being part of a CS group is helping to develop my self-awareness
	10. I have gained new clinical insights through supervision
	11. Clinical supervision has made me more aware of areas of skill I need to improve
	12. Clinical supervision has definitely had a positive impact on the quality of care I provide
	13. Clinical supervision has helped me cope with any stresses at work I may have
	14. Clinical supervision has helped me feel more confident about dealing with my job

### Translation procedure

The original instrument in English [8] was first translated into Swedish separately by the three authors. These versions were compared, and the three translation sets were compared, discussed and synthesized to form a fourth set. The Swedish version was then sent to a blinded bilingual professional translator for back-translation. This revealed some minor discrepancies compared to the original scale, and alterations were made in dialogue with the bilingual translator to ensure that the original meaning of every item was kept intact during the translation process. Six Swedish-speaking university lecturers were cognitively interviewed and systematically debriefed to ensure a semantic review of the wording of the items in Swedish in connection with the content validity evaluation. No linguistic flaws were pointed out.

### Data collection

Data for the calculation of content validity was collected from an expert group of six university lecturers with experience and knowledge of clinical supervision and reflection in groups. The experts were asked to rate the relevance of each item of the scale on a four-point Likert scale. Each item was rated on a four-point Likert scale where 1 connoted an irrelevant item and 4 connoted a highly relevant item.

Data for the reliability analysis was collected from rehabilitation assistants and unit managers participating in RPGs in the beginning of the intervention period at the second group session. All participants (*n* = 20) except one agreed to fill out the Swedish version of the survey. Questionnaires were also distributed at later sessions to evaluate the RPGs. Data from later sessions are not included in this analysis.

### Data analysis

According to Polit and Beck [22], content validity pertains to the degree in which an instrument has an appropriate sample of items for the construct being measured and whether or not the items adequately represent the domain of content. Content validity was measured by computing a content validity index (CVI), following the process described by Polit and Beck [22]. The experts’ ratings of content relevance were measured on a four-point Likert scale. According to Polit and Beck [22], a rating of 1 or 2 indicates deficits in content validity, whereas a rating of 3 or 4 indicates that the item is content valid. The ratings were dichotomized into two groups indicating irrelevance (values 1–2) or content validity (values 3–4). The average CVI for each item (I-CVI) was computed by taking the number of experts deeming the item as content valid divided by the total number of experts. This generated an I-CVI for each item, and the average CVI for the scale as a whole was computed by computing the average CVI of all I-CVIs.

Construct validity was assessed by calculating the corrected item-total correlation statistics. Correlation values > 0.20 were considered satisfactory, in accordance with the values proposed by Kline [23].

Reliability was evaluated by analysing the Cronbach’s alpha coefficient, the intraclass correlation coefficient (ICC) and inter-item correlations. According to Nunnally [24], a Cronbach’s alpha value above > 0.70 is considered satisfactory.

## Results

### Sample

The mean age in the sample was 48 years, and gender distribution was even. Mean years of experience in the healthcare sector was 18.9 years and 9.9 years in psychiatric care. Most respondents were educated psychiatric nursing assistants and worked mostly daytime shifts (Table 2).

**Table 2:** Characteristics of the study sample

	Respondents *n* = 20
Age m(sd)	
m (SD)	48 (11.2)
Range (min-max)	33 (27–60)
Gender n (%)	
male	10 (50%)
female	10 (50%)
Years of experience m(sd)	
in healthcare	18.9 (10.1)
in psychiatric care	9.9 (9.1)
Education n (%)^a^	
no formal education in healthcare	1 (5.3%)
nursing assistant	2 (10.5%)
psychiatric nursing assistant	13 (68.4%)
other relevant education	3 (15.8%)
Works n (%)	
mostly day	17 (85%)
mostly night	3 (15%)

^a^data from one respondent is missing

### Validity

#### Content validity

The average CVI for each item (I-CVI) is presented in Table 2. The average CVI for the scale as a whole (S-CVI) was 0.94, indicating good content validity. However, item number 5, ‘*There are well-established ground rules in my group,’* demonstrated poor content validity (I-CVI < 0.5).

#### Construct validity

Item-total correlations ranged between 0.23 and 0.81, and deletion of any item did not notably improve Cronbach’s alpha (Table 3).

**Table 3:** Item statistics

	Corrected Item-Total Correlation	Cronbach’s Alpha if Item Deleted	I-CVI	Minimum	Maximum	Mean	Std. Deviation
Item1	0.70	0.88	1	1.00	2.00	1.95	0.22
Item2	0.23	0.89	1	1.00	2.00	1.80	0.41
Item3	0.51	0.88	0.83	1.00	2.00	1.55	0.51
Item4	0.73	0.87	1	0.00	2.00	1.80	0.52
Item5	0.51	0.88	0.5	−1.00	2.00	1.40	0.82
Item6	0.81	0.86	1	−1.00	2.00	1.70	0.80
Item7	0.55	0.88	0.83	− 1.00	2.00	1.40	0.82
Item8	0.73	0.87	1	0.00	2.00	1.80	0.52
Item9	0.39	0.88	1	0.00	2.00	1.55	0.60
Item10	0.38	0.88	1	1.00	2.00	1.55	0.51
Item11	0.37	0.88	1	1.00	2.00	1.20	0.41
Item12	0.63	0.87	1	0.00	2.00	1.10	0.72
Item13	0.80	0.86	1	−1.00	2.00	0.80	0.70
Item14	0.65	0.87	1	0.00	2.00	1.10	0.72

### Reliability

Cronbach’s alpha for the scale was 0.89. A two-way mixed effects model for calculating the ICC was used. The ICC for single measures was 0.35 (CI95 0.21–0.56). The inter-item correlation matrix (Table 4) revealed that many items correlated below .30, and some items correlated over .70. The mean inter-item correlation was .37.

**Table 4:** Inter-Item Correlation Matrix

	Item1	Item2	Item3	Item4	Item5	Item6	Item7	Item8	Item9	Item10	Item11	Item12	Item13	Item14
Item1														
Item2	0.46													
Item3	0.25	0.30												
Item4	0.36	0.29	0.43											
Item5	0.69	0.25	0.20	0.20										
Item6	0.79	0.29	0.42	0.73	0.51									
Item7	0.69	0.09	0.08	0.32	0.61	0.67								
Item8	0.36	0.29	0.43	1.00	0.20	0.73	0.32							
Item9	0.21	0.25	0.16	0.70	0.06	0.47	0.17	0.70						
Item10	0.25	0.05	0.19	0.43	0.08	0.30	−0.05	0.43	0.33					
Item11	0.11	−0.06	0.45	0.20	0.22	0.19	0.22	0.20	0.17	0.20				
Item12	0.36	0.07	0.42	0.48	0.38	0.42	0.29	0.48	0.11	0.42	0.29			
Item13	0.61	0.04	0.47	0.46	0.61	0.64	0.70	0.46	0.15	0.18	0.52	0.78		
Item14	0.36	−0.11	0.56	0.48	0.29	0.51	0.38	0.48	0.11	0.56	0.29	0.69	0.67	
*	8/13	1/13	8/13	8/13	5/13	8/13	5/13	7/13	4/13	3/13	8/13	9/13	9/13	9/13
**	0.42	0.15	0.35	0.48	0.30	0.49	0.32	0.48	0.28	0.26	0.24	0.40	0.47	0.41

* The proportion of times the item correlates between .30 and .70 with other items in the scale

** The average inter-item correlation for each item

## Discussion

The internal consistency reliability was measured by the corrected item-total correlations. Item-total correlations ranged between 0.23 and 0.81, in line with the standards recommended by Kline [23] and comparable to the results obtained for the original version of the instrument in English [11]. This indicates that items varied in line with each other and that each item was consistent with the averaged behaviour of the other items. Cronbach’s alpha for the scale was 0.89, indicating good internal consistency according to the standards described by Nunnally [24]. Horton et al. [8] evaluated the English version of the CSEQ and found the instrument to have good validity and reliability. They found a Cronbach’s alpha of 0.86, which is close to the alpha of 0.89 that was found in our study. Kuipers et al. [25] found a Cronbach’s alpha of 0.93 for the English version of the scale.

The ICC for single measures was 0.35, indicating low resemblance within the items in the instrument. When the variance between respondents is low, the ICC is expected to be low as well [24]. Many inter-item correlations were below .30, indicating that they are not sufficiently related and therefore do not contribute to the measurement of the core factor. Some correlations were above .70, indicating redundancy. Low correlations were expected, as the original instrument is divided into three factors; *aim*, *process* and *effects*. The more the items in a scale resemble each other, the more they measure the same attribute. Our findings indicate heterogeneity in the instrument. A possible solution to increase homogeneity is to decrease the number of items in the scale. However, this may reduce instrument sensitivity [26].

Construct validity is assessed on the basis of correlations from numerous studies where the instrument is used and evaluated. Kuipers et al. [25] used the CSEQ to evaluate the outcome of clinical supervision and found that the scale and its subscales demonstrated good internal consistency. They found alpha values of 0.93 for the instrument in total, and for the subscales, they found alpha values of 0.76 for the Purpose subscale, 0.95 for the Process subscale, and 0.91 for the Impact subscale. Horton et al. [8] found the convergent validity of the CESQ by asking participants about their general opinions of the clinical supervision program and found a significant correlation coefficient of 0.79 with the overall CSEQ score.

Content validity for the scale was high, except item number five. This item proved rather difficult to translate. To translate the item without violating the original meaning there, we decided to translate in accordance with meaning rather than exact wording. This may have clouded the connection of the item to the construct under study, thus influencing content validity negatively. The translation process was according to the standards given by Maneesriwongul and Dixon [27], including translation and back-translation using a professional bilingual translator. Still, deficits in the translation might have contributed to the heterogeneity identified in the inter-item correlations and the low rating of content validity for item number 5. Therefore, we suggest that the wording of item number 5 be revised before the instrument is used in a clinical context.

The instrument is tested in the context it is aimed for, and the participation rate in this study was 95.2%, with only one member of the clinical supervision group declining the opportunity to complete the instrument. However, the sample was small (*n* = 20). According to Ferketish [26], the sample should be at least five times as many as the items in the instrument. Such a small sample of participants implies that the reliability of the study findings can be questioned. This calls for the need to further study the psychometric properties of the S-CSEQ in a larger sample. However our findings are coherent with other psychometric evaluations of the original version of the CSEQ.

A test-retest of instrument reliability was not performed because the instrument is not possible to test outside a clinical supervision group, and because thereflective process within a clinical supervision group is bound to influence participants during the test-retest period.

## Conclusion

Our findings provide initial support that the S-CSEQ demonstrates acceptable reliability and validity in the mental health context. Our results are similar to the results from psychometric evaluations of the English version of the instrument. Reliability analyses demonstrated good internal consistency of the instrument, although some heterogeneity in the instrument was found. Validity analyses revealed good construct validity, and content validity was good for all items except item number five. We therefore suggest that the wording of item five be revised before the instrument is used in a clinical context. Our findings indicate that the S-CSEQ has a sufficiently high degree of reliability and validity to be used as an assessment of RPGs in the mental health context, although further psychometric analyses with a larger sample are recommended.

## Abbreviations

CSEQ: Clinical Supervision Evaluation Scale
CVI: Content Validity Index
ICC: Intraclass Correlation Coefficient
RPG: Reflective Practice Group
S-CSEQ: The Swedish Version of the Clinical Supervision Evaluation Scale
